# Efficacy and safety of immune checkpoint inhibitors combined with chemotherapy as first-line treatment for extensive-stage small cell lung cancer: a meta-analysis based on mixed-effect models

**DOI:** 10.3389/fmed.2023.1198950

**Published:** 2023-07-31

**Authors:** Jianqing Zheng, Yujie Deng, Bifen Huang, Xiaohui Chen

**Affiliations:** ^1^Department of Radiation Oncology, The Second Affiliated Hospital of Fujian Medical University, Quanzhou, Fujian, China; ^2^Department of Medical Oncology, The First Affiliated Hospital of Fujian Medical University, Fuzhou, Fujian, China; ^3^Department of Obstetrics and Gynecology, Quanzhou Medical College People's Hospital Affiliated, Quanzhou, Fujian, China; ^4^Department of Thoracic Surgery, Fujian Cancer Hospital, Clinical Oncology School of Fujian Medical University, Fuzhou, Fujian, China; ^5^The Graduate School of Fujian Medical University, Fuzhou, China; ^6^Fujian Key Laboratory of Advanced Technology for Cancer Screening and Early Diagnosis, Fuzhou, China

**Keywords:** extensive-stage small cell lung cancer, immune checkpoint inhibitors, chemotherapy, programmed death-ligand 1, first-line treatment

## Abstract

**Background:**

Extensive-stage small cell lung cancer (ES-SCLC) is a highly invasive and fatal disease with limited therapeutic options and poor prognosis. Our study aims to systematically evaluate the efficacy and safety of immune checkpoint inhibitors combined with chemotherapy (ICIs+ChT) vs. chemotherapy alone (ChT) in the first-line treatment of ES-SCLC.

**Methods:**

A literature search was performed for randomized controlled trials (RCTs) related to “ICIs+ChT” vs. “ChT” in the first-line treatment of ES-SCLC in PubMed, Cochrane Library, Embase, CNKI, and other databases. RevMan 5.4 software was used to perform meta-analyses with hazard ratio (HR) and relative risk (RR). SAS 9.4 software was applied to conduct a mixed-effect model meta-analysis of the survival outcomes and draw survival curves.

**Results:**

A total of 2,638 patients with ES-SCLC from 6 RCTs were included, of which 1,341 patients received “ICIs+ChT” and 1,297 received ChT. Based on the meta-analysis results provided by the mixed-effect model, patients receiving the “ICIs+ChT” regimen had a significantly longer overall survival (OS, HR = 0.800, 95% CI = 0.731–0.876, *P* < 0.001) and progression-free survival (PFS, HR = 0.815, 95% CI = 0.757–0.878, *P* <0.001) in comparison to those receiving ChT only. Compared with ChT, “ICIs+ChT” did neither improve the objective response rate (ORR, RR = 1.06, 95% CI = 1.00–1.12, *P* = 0.06) nor did it improve the disease control rate (DCR, RR = 0.97, 95% CI = 0.92–1.03, *P* = 0.35). Although the incidence of grade 3 to 5 treatment-related adverse events (trAEs) in the “ICIs+ChT” subgroup did not increase (RR = 1.16, 95% CI = 0.97–1.39, *P* = 0.11), the incidence of grade 3 to 5 immune-related adverse events (irAEs) increased significantly (RR = 4.29, 95% CI = 1.73–10.61, *P* < 0.00001).

**Conclusion:**

ICIs+ChT regimen could significantly prolong OS and PFS in patients with ES-SCLC compared with ChT alone. Although the incidence of irAEs in “ICIs+ChT” is higher than that in the “ChT” subgroup, the incidence of trAEs is similar within the two subgroups. ICIs combined with chemotherapy demonstrated a good choice as first-line treatment for ES-SCLC.

**Systematic review registration:**

PROSPERO, identifier: CRD42022348496.

## 1. Introduction

Small cell lung cancer (SCLC) is a neuroendocrine tumor (NET) originating from argyrophilic cells of bronchial mucosal epithelium or glandular epithelium, which is a highly malignant form and accounts for approximately 15–20% of all lung cancers (1). SCLC has completely different molecular markers and biological behaviors from non-small cell lung cancer (NSCLC) (2). Clinically, SCLC is subdivided into extensive-stage (ES-SCLC) and limited-stage (LS-SCLC) diseases, accounting for 60–70% and 30–40% of diagnosed SCLC, respectively (1). The cisplatin-based doublet chemotherapy (ChT), i.e., etoposide/irinotecan plus cisplatin/carboplatin (EP/IP regimen), has been used as standard initial (first-line) treatment of ES-SCLC for decades, with an objective response rate (ORR) of <60% (3). Although SCLC has a unique sensitivity to chemoradiotherapy, the 5-year overall survival (OS) rate is still <5% in ES-SCLC patients receiving standard first-line treatment (4). ES-SCLC patients suffering from recurrence or progression during or shortly after initial treatment are usually embarrassed with limited therapeutic options and poor prognosis (5). With the continuous in-depth study of immune checkpoint inhibitors (ICIs) and immunotherapy, a couple of randomized phase III trials demonstrated that a combination of atezolizumab (IMpower133) (6) or durvalumab (CASPIAN) (7, 8) with EP could improve OS in ES-SCLC patients. Therefore, the combined regimen had been approved as the first-line treatment for ES-SCLC patients, establishing a milestone in the management of SCLC (9). However, not all ICIs exhibited better anti-tumor activity in the first-line treatment of ES-SCLC. The CA184-156 study confirmed that adding ipilimumab to EP would not improve the OS of ES-SCLC patients (10). Nivolumab and pembrolizumab were first approved by the FDA as third-line treatments for ES-SCLC, but recent studies had indicated that the combination of EP with either nivolumab or pembrolizumab as the first-line treatment can significantly improve the OS and PFS of ES-SCLC.

In this study, we conducted a systematic review of the randomized controlled trials published recently to evaluate the “ICIs+ChT” regimen as the first-line treatment of ES-SCLC, aiming to evaluate the abovementioned evidence objectively based on the principles and methods of evidence-based medicine (EBM) and the GRADE criteria developed by the Grading of Recommendations Assessment, Development and Evaluation Working Group. The future development direction of this field was also discussed, with a view to providing evidence that is more in line with the requirements of EBM for the first-line treatment of ICIs for ES-SCLC.

## 2. Materials and methods

### 2.1. Study protocol

The current study was conducted according to the Preferred Reporting Items for Systematic Reviews and Meta-analyses (PRISMA) (11), and the quality control and quality assurance (QC and QA) of the manuscript were instructed by the corresponding authors (Jianqing Zheng and Xiaohui Chen). The review was prospectively registered on PROSPERO (CRD42022348496).

### 2.2. Literature inclusion criteria

#### 2.2.1. Study design

Randomized controlled trials (RCTs) of “ICIs+ChT” vs. “ChT” in the first-line treatment of ES-SCLC were recruited, regardless of whether the blind method is used, and the language is not limited.

#### 2.2.2. Study participants

The study participants are those (1) who had SCLC diagnosed and confirmed by pathology; (2) who were diagnosed as extensive stage according to SCLC staging criteria proposed by the Veterans Administration Lung Study Group (VALG), and some recurrent SCLC are not restricted; and (3) patients who have not received any other first-line treatments in the past, including chemotherapy and targeted therapy.

#### 2.2.3. Interventions

Interventions include (1) conventional chemotherapy in the control group. However, the chemotherapy regimen and chemotherapy cycle were not limited; and (2) ICIs alone or in combination with chemotherapy in the experimental group, and other clinical treatments were the same as those in the control group.

#### 2.2.4. Outcomes

(1) The primary outcomes are OS and PFS. To achieve a meta-analysis based on the linear mixed-effect models, the survival proportions of OS and PFS were also extracted from the survival curves. (2) The secondary outcomes were ORR and DCR. (3) The incidence of grade 3 to 5 adverse events: According to 1988 WHO anti-cancer drug side effects standard or common adverse event evaluation standard CTCEA version 4.0, adverse events were further subdivided into treatment-related adverse events (trAEs) and immune-related adverse events (irAEs).

### 2.3. Literature exclusion criteria

The exclusion criteria included (1) studies involving non-clinical trials or non-RCTs; (2) research with incomplete data or the relevant data could not be extracted; (3) repeated publications or serial publications with the latest literature; (4) studies involving patients that received any other first-line treatments in the past, such as chemotherapy combined with irradiation and molecular targeted therapy.

### 2.4. Search strategies

#### 2.4.1. Database

A comprehensive literature search on the PubMed, Cochrane Library databases Embase, and CNKI was performed, covering all publications in these databases up to 1 February 2021.

#### 2.4.2. Search terms

(1) Search terms related to disease were Small Cell Lung Cancer, Small Cell Cancer of The Lung, Oat Cell Lung Cancer, Small Cell Lung Tumor, Small Cell Lung Neoplasm, Carcinoma, Small Cell Lung, etc. (2) Search terms related to drugs or immunotherapy were Ipilimumab, Pembrolizumab, Nivolumab, Atezolizumab, Durvalumab, and other ICIs. The trade name of the drug includes Yervoy, Keytruda, Opdivo, Tecentriq, Imfinzi, etc. (3) Other search terms included anti-CTLA-4 mAb, anti-PD-L1, anti-PD-1, PD-1 Receptor, Programmed Cell Death 1 Protein, PD-1, PD-L1, etc.

#### 2.4.3. Retrieval strategies

Combined with the RCTs filter of the database, the subject terms with free words were applied to conduct a preliminary retrieval of the literature and the reviews, case reports, meta-analysis, and other types of literature were filtered out. Independent searches were conducted by two investigators (first co-authors) in accordance with the abovementioned search principles. When there was a disagreement, the third investigator (corresponding author) will be consulted. Further manual and electronic database searches were carried out using the reference lists attached to the eligible articles. At the same time, search engines, such as Google Scholar, were used to find relevant literature on the Internet and to trace the references that had been included in the literature, in order to expand the scope of retrieval.

### 2.5. Literature extraction and quality assessment

#### 2.5.1. Literature extraction

Two independent researchers reviewed and evaluated the title and abstract of each RCT according to the determined search strategies, and the potentially eligible articles that meet the selection criteria would be recruited. After discussion in accordance with the inclusion criteria, literature extraction was performed, a consensus was reached, and a decision was made to finally include or exclude the eligible articles. If a consensus could not be met, the corresponding author of this article was responsible for the final ruling.

#### 2.5.2. Quality assessment

Two independent researchers evaluated the included RCTs according to the bias risk assessment method recommended by the Cochrane Assistance Network. The evaluation methodological criteria and items were as follows: (1) generation of random allocation sequence; (2) the method of allocation concealment; (3) the method of blinding the patients; (4) the method of blinding the doctors or the therapists; (5) the method of blinding the data collectors and analysis personnel; (6) incomplete data reported; (7) selective reporting bias; and (8) other potential bias affecting authenticity.

We evaluated the risk of bias for each RCT according to the following criteria: “Yes” indicates a low risk of bias; “No” indicates a high risk of bias; and “Unclear” indicates that the literature does not provide sufficient information for bias assessment. The two researchers discussed according to the abovementioned standards and methods and reached a consensus according to the opinions of the third researcher.

#### 2.5.3. Assessment of the grade of recommendation and the level of evidence

Overall quality and level of recommendation of evidence were evaluated based on the results of the systematic review. The GRADE system was used to evaluate the quality of evidence (12). Quality of evidence is graded as follows: (1) high quality: further research is unlikely to change our confidence in the estimate of effect; (2) moderate quality: further research is likely to have an important impact on our confidence in the estimate of effect and may change the estimate; (3) low quality: further research is very likely to have an important impact on our confidence in the estimate of effect and is likely to change the estimate; (4) very low quality: we have very little confidence in the effect estimate: the true effect is likely to be substantially different from the estimate of effect. Although the evidence based on RCT was initially rated as high quality, our confidence in this type of evidence may be reduced due to the following five factors: (1) limitations of the research; (2) inconsistent results; (3) indirect evidence; (4) inaccurate results; and (5) biased results. Finally, the GRADEpro software was used to edit, analyze, and map the evidence grade.

### 2.6. Data extraction

After reading the full text, two researchers extracted and cross-checked the data, including (1) basic information, such as the title of the trial, author's name, year of publication, and source of literature; (2) methodological information about the trial: the sample size of the study included the basic information of the study population, including the entry time, the number of participants, disease stages, the randomization method of the trial, the evaluation method of important outcome indicators, median follow-up duration, death, and withdrawal; (3) detailed information on following intervention measures: ICI medication and medication in the control group; (4) outcome indicators: survival proportions information in the survival curve, HR for OS and PFS with corresponding 95% CIs, ORR and DCR information, and the incidence of related adverse events. Disagreements were resolved by consensus.

### 2.7. Statistical analysis

For time-survival variables (such as OS and PFS), HR and its corresponding 95% CIs were applied as the effect size. If HR and its 95% CI could not be obtained from the trials directly, they were extracted according to the method introduced by Parmar et al. (13). For binary variables (such as ORR, DCR, and AEs), the relative ratio (RR) and its 95% CI were used as the effect size. RevMan 5.4 software was used to conduct quantitative and comprehensive analyses. The chi-square test (χ^2^ test) was applied to determine whether there was heterogeneity within studies, and the index I^2^ (range, 0–100%) was selected to measure the degree of heterogeneity within studies. The index I^2^ ≥ 50% or *P*-value of χ^2^ test <0.1 indicated significant heterogeneity within studies. If there was no statistical heterogeneity, the fixed effects model would be used; if not, the random effects model was used to perform the meta-analysis. If the source of heterogeneity cannot be judged or the data provided by the trials cannot be used for meta-analysis, descriptive analysis would be used.

For survival variables, an additional method based on the mixed-effect model, which was described by Arends et al. (14), was applied to perform a meta-analysis on the survival rate in the survival curves, and some interesting summary survival curves were drawn in this study. Statistical analysis was performed using SAS 9.4 software.

The quality evaluation based on the GRADE system was graded and mapped via GRADE pro 3.6 software. Two independent reviewers rated the evidence based on the quality of the evidence and the subject of the study. If there was a dispute, a third reviewer would be asked to meet a consensus by means of panel discussion.

## 3. Results

### 3.1. Literature search results

**Figure 1:**
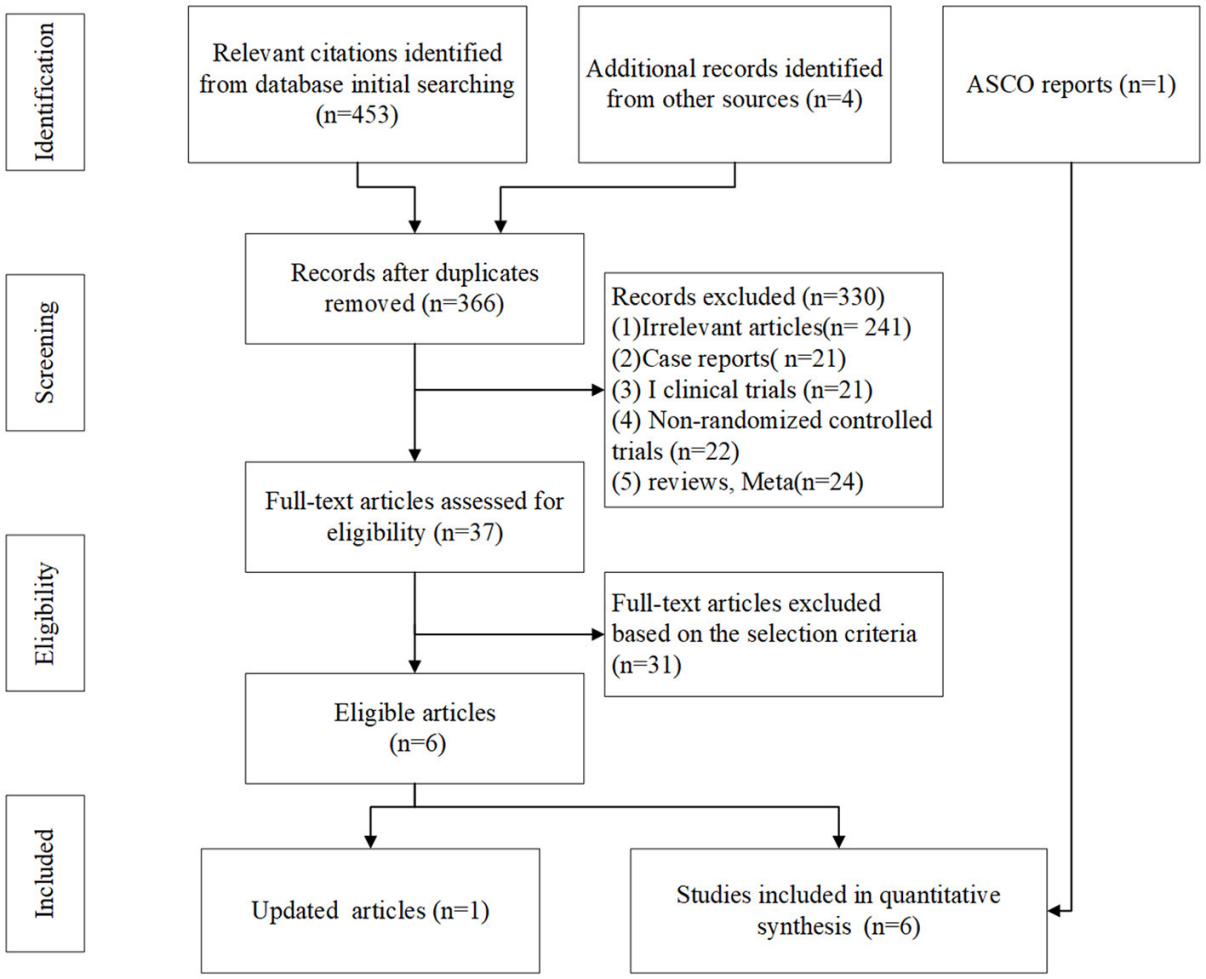
Study selection flow chart of the meta-analysis.

In total, 459 related literature were initially detected, among which 93 were identified as duplicates and then removed. From the remaining 366, 329 articles were found to be published repeatedly and obviously did not meet our inclusion criteria. After intensively reviewing the titles and abstracts, we identified 37 controlled clinical studies. After further searching and reviewing the full text, we eventually enrolled six RCTs after excluding clinical trials that were inconsistent with our inclusion and exclusion criteria. Those six RCTs were as follows: NCT00527735 (15), NCT01450761 (CA184-156) (10), NCT02763579 (IMpower133) (6), NCT03043872 (CASPIAN) (7, 8), NCT03066778 (KEYNOTE-604) (16), and NCT03382561 (ECOG-ACRIN EA5161) (17). A total of seven articles reported the above six clinical trials, of which ECOG-ACRIN EA5161 was reported at ASCO in 2020. The literature screening process is shown in Figure 1.

### 3.2. General information of the included studies

A total of 2,638 SCLC patients were included, of which 1,297 patients received chemotherapy alone and 1,341 received “ICIs+ChT” (immunotherapy group). Two studies involved ipilimumab (10, 15), and other studies involved atezolizumab, durvalumab, pembrolizumab, and nivolumab. Six studies were conducted with “ICIs+ChT” vs. “ChT” alone, of which one was a three-arm trial (15) and two were phase II RCTs (15, 17). The basic characteristics of included studies are shown in Table 1.

**Table 1:** Characteristics of the analyzed trials.

**References**	**Trial name**	**Phase**	**Treatment**	**Sample**	**Gender/male**	**Median age**	**ECOG score/0**
Reck et al. (15)	NCT00527735	II	C/IC/IC	45/43/43	33/33/32	58/57/59	12/8/11
Reck et al. (10)	CA184-156	III	C/IC	476/478	326/317	63/62	147/137
Horn et al. (6)	Impower133	III	C/AC	202/201	132/129	64/64	67/73
Paz-Ares et al. (8); Goldman et al. (7)	CASPIAN	III	C/DC	269/268	184/190	63/62	90/99
Rudin et al. (16)	KEYNOTE-604	III	C/PC	225/228	142/152	65/64	56/60
Leal et al. (17)	ECOG-ACRIN EA5161	II	C/NC	80/80	44/45	65/65	24/23

C, chemotherapy; I, ipilimumab; A, atezolizumab; D, durvalumab; N, nivolumab; P, pembrolizumab.

### 3.3. Risk of bias assessment of included studies

**Figure 2:**
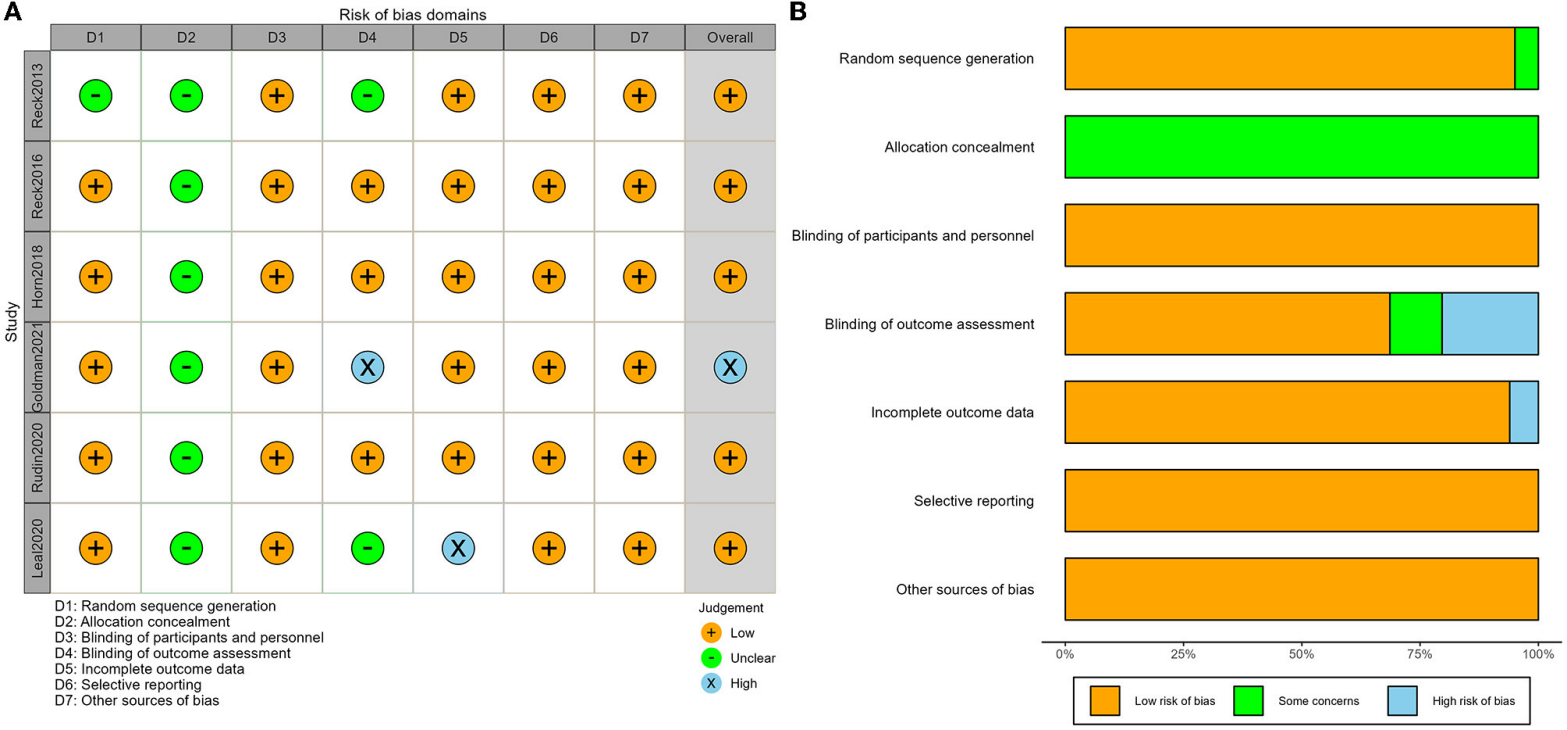
Risk of bias assessment of included studies: **(A)** graph of risk of bias and **(B)** summary of risk bias.

According to the Cochrane Collaboration tool for assessing the risk of bias of RCTs, five of the six studies had explicitly mentioned the random allocation method, while one study did not specify it (15). All the studies did not mention the detailed method of allocation concealment. Outcome-rater blinding was applied and described in detail in three studies (6, 10, 16), was unspecified in two studies (15, 17), and was not applied in one study (7). One study lacked information on DCR and was considered incomplete outcome data (17). All studies were judged free from selection reporting bias and were judged free from other biases. The risk of bias for each study is shown in Figure 2A, and the risk of bias for the overall study is shown in Figure 2B, with yellow for low risk of bias, green for unclear risk of bias, and blue for high risk of bias. Therefore, the six studies included in this article were all of high quality.

### 3.4. Classification of quality of evidence and strength of recommendation sistema grade

**Figure 3:**
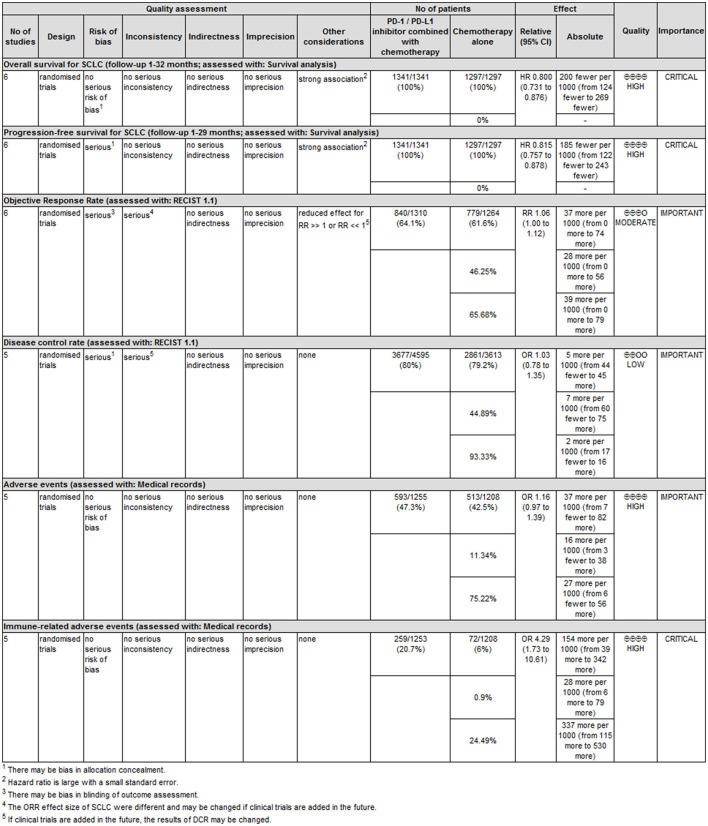
GRADE evidence profile for main outcomes.

There were six primary outcomes in this study, namely OS, PFS, ORR, DCR, and the incidence of trAEs and irAEs as well. OS, PFS, and the incidence of irAEs were set as “key outcomes” and others as “important outcomes”. The classification of the quality of evidence and the strength of recommendation *via* the GRADE system are shown in Figure 3 with detailed reasons for upgrading or downgrading the quality of evidence for each outcome.

### 3.5. Meta-analysis of overall survival

**Figure 4:**
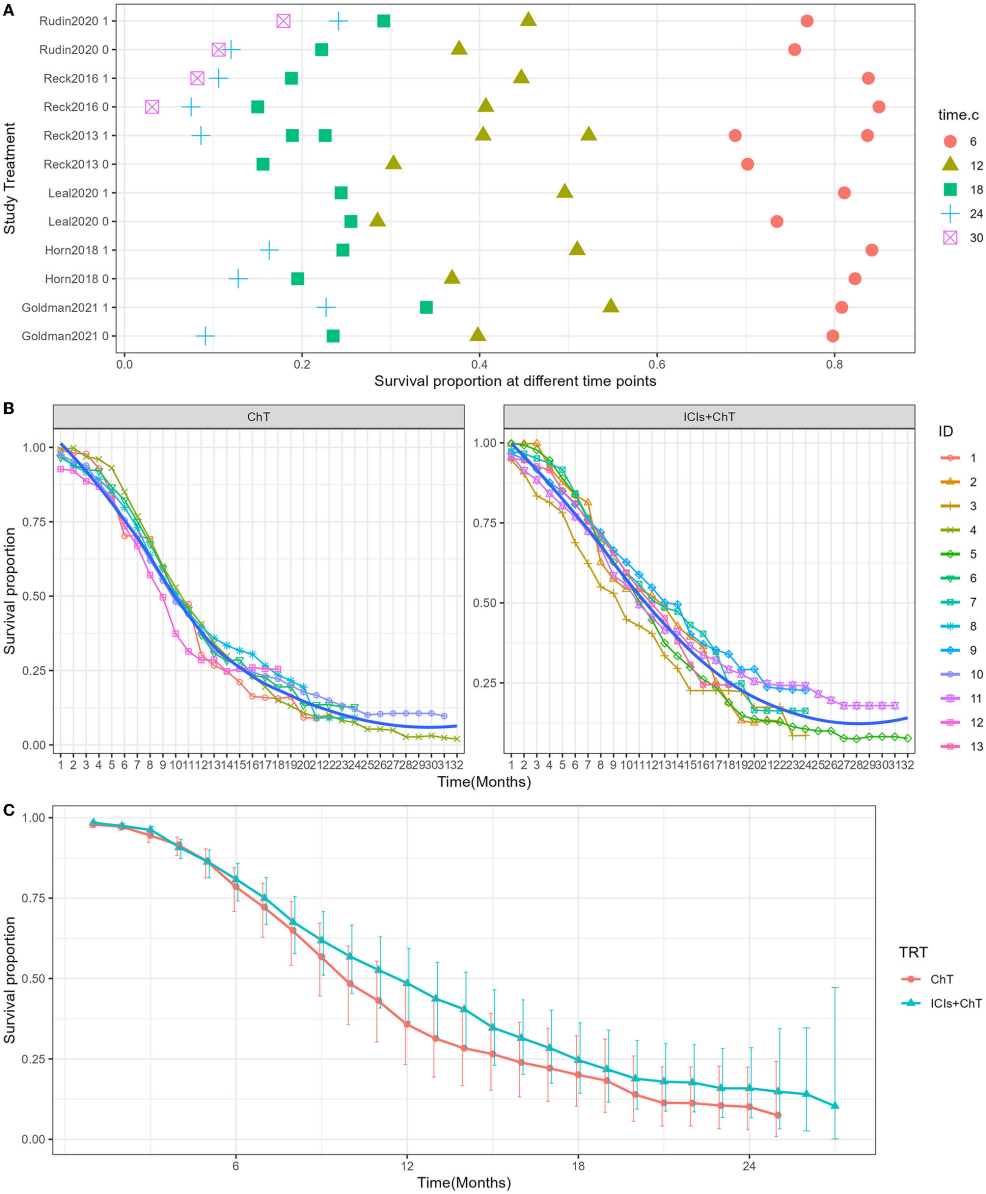
Meta-analysis results of overall survival for SCLC: **(A)** overall survival proportions distribution chart; **(B)** overall survival curves for SCLC paneled by different treatments (trts, ChT: chemotherapy, ICIs+ChT: immune checkpoint inhibitors+ chemotherapy); **(C)** grouped meta-analyzed overall survival curves with error bars.

Survival curves had been provided in all studies, and the survival proportions at each time point were successfully extracted in all included studies. The detailed distribution of OS proportions is shown in Figure 4A. Reconstructed survival curves based on the extracted survival proportions are shown in Figure 4B and are paneled by different treatments (trts).

**Figure 5:**
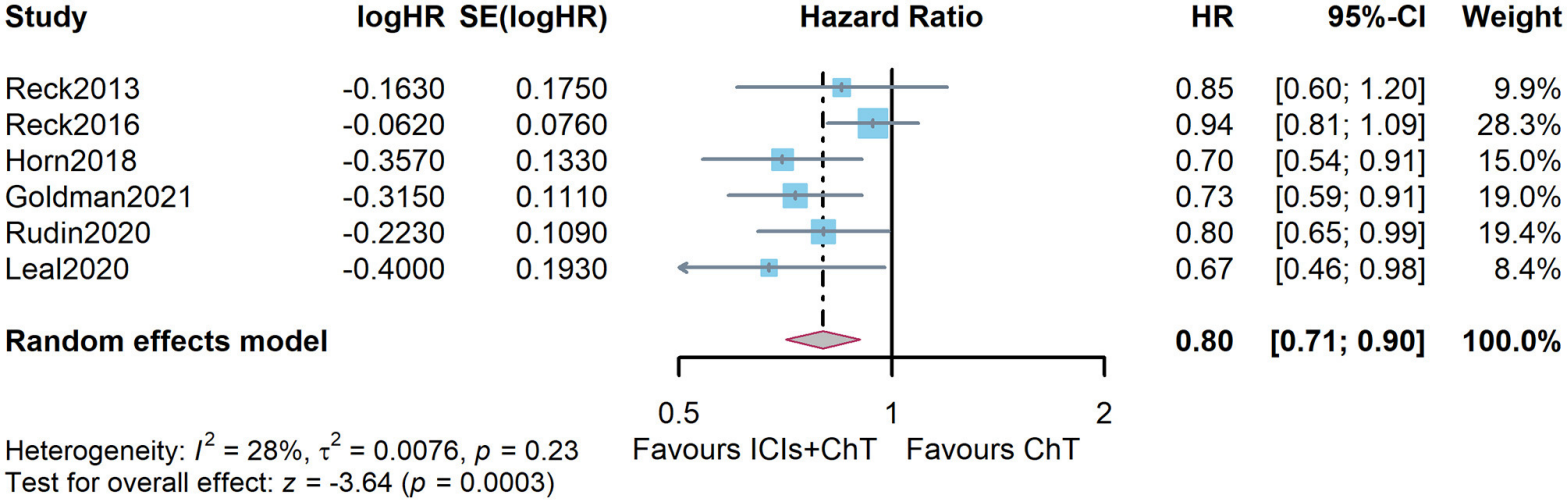
Comparison of overall survival (OS) between ICIs+ ChT and ChT based on the traditional hazard ratio model.

Figure 4C shows the meta-analysis results of OS based on the extracted survival proportions *via* the mixed-effect model. The statistical effect value of the difference in survival between the two groups was HR = 0.800, 95% CI = 0.731–0.876, and *P* < 0.001. The summary survival proportions at each time point are shown in Table 2. For the convenience of comparison, the meta-analysis results based on the traditional hazard ratio model are shown in Figure 5. Meta-analysis effect size of OS was HR = 0.82, 95% CI = 0.75–0.90, and *P* < 0.00001.

**Table 2:** Grouped meta-analyzed survival proportions for SCLC (%).

**Group**	**Time point (month)**	**OS (95% CI)**	**PFS (95% CI)**
ChT	6	78.58% (95% CI = 70.89–84.47)	26.26% (95% CI = 15.57–38.24)
ICIs +ChT	6	80.75% (95% CI = 74.14–85.83)	35.95% (95% CI = 24.94–47.06)
ChT	12	35.8% (95% CI = 23.26–48.5)	4.82% (95% CI = 1.47–11.29)
ICIs +ChT	12	48.46% (95% CI = 36.55–59.38)	12.23% (95% CI = 5.77–21.25)
ChT	18	20.07% (95% CI = 10.29–32.17)	-
ICIs +ChT	18	24.57% (95% CI = 14.36–36.23)	-
ChT	24	10.12% (95% CI = 2.98–22.45)	-
ICIs +ChT	24	15.84% (95% CI = 6.7–28.48)	-

### 3.6. Meta-analysis of progression-free survival

**Figure 6:**
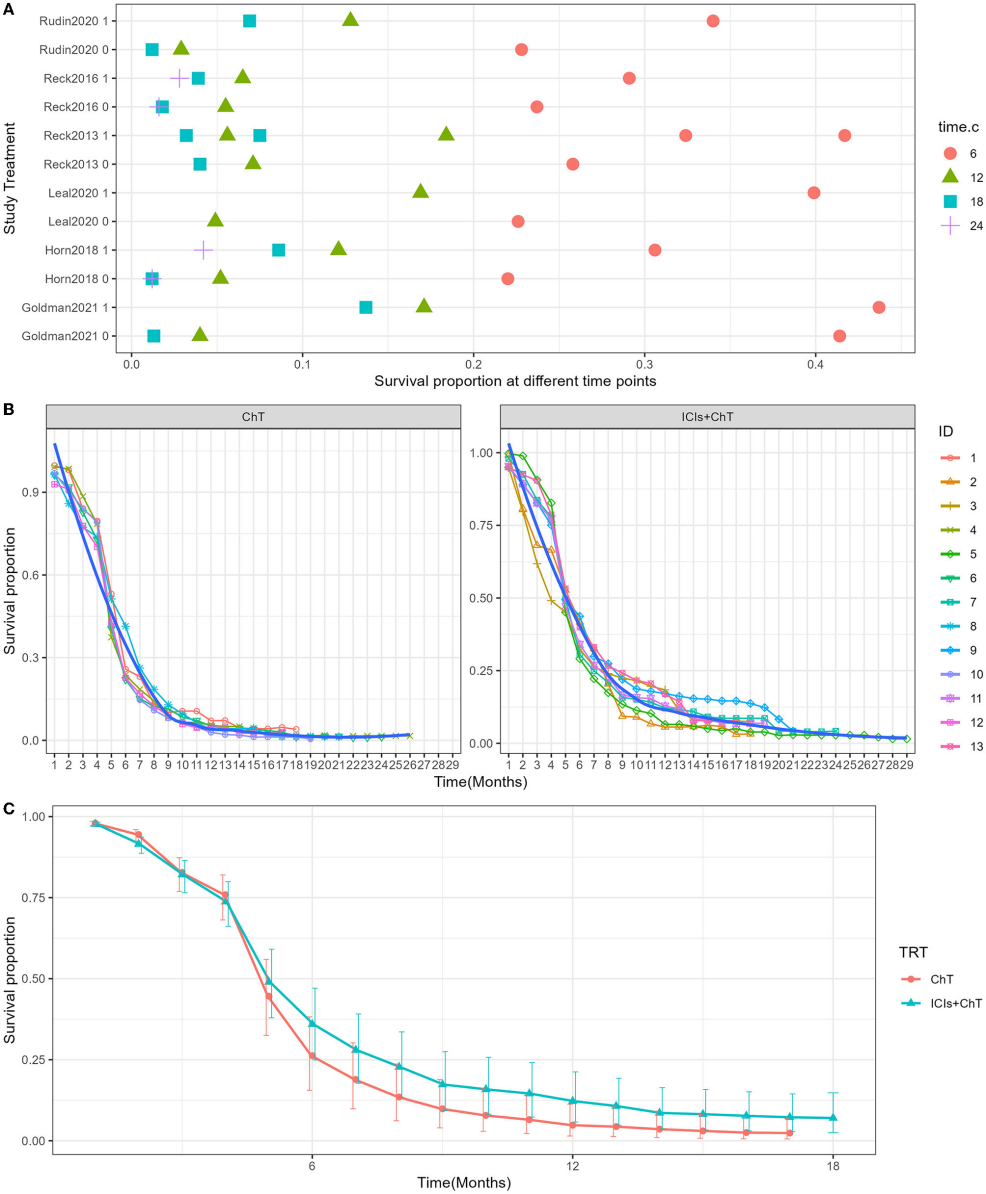
Meta-analysis results of progression-free survival for SCLC: **(A)** Progression-free survival proportions distribution chart; **(B)** Progression-free survival curves for SCLC paneled by different treatments (trts, ChT: chemotherapy, ICIs+ChT: immune checkpoint inhibitors+ chemotherapy); **(C)** grouped meta-analyzed progression-free survival curves with error bars.

The detailed distribution of PFS proportions is shown in Figure 6A. Reconstructed PFS curves based on the extracted survival proportions are shown in Figure 6B and are paneled by different treatments (trts).

**Figure 7:**
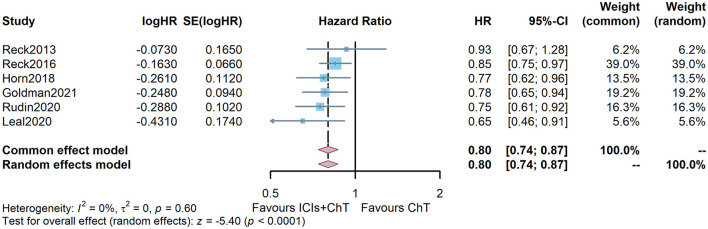
Comparison of progression-free survival (PFS) between ICIs+ChT and ChT in SCLC.

Figure 6C shows the meta-analysis results of PFS based on the extracted survival proportions *via* the mixed-effect model. The statistical effect values of the difference in survival between the two groups were HR = 0.815, 95% CI = 0.757–0.878, and *P* <0.001. The summary survival proportions at each time point are shown in Table 2. The meta-analysis results based on the traditional hazard ratio model are shown in Figure 7. The meta-analysis effect size of PFS was HR = 0.80, 95% CI = 0.74–0.87, and *P* < 0.00001.

### 3.7. Meta-analysis of ORR and DCR

**Figure 8:**
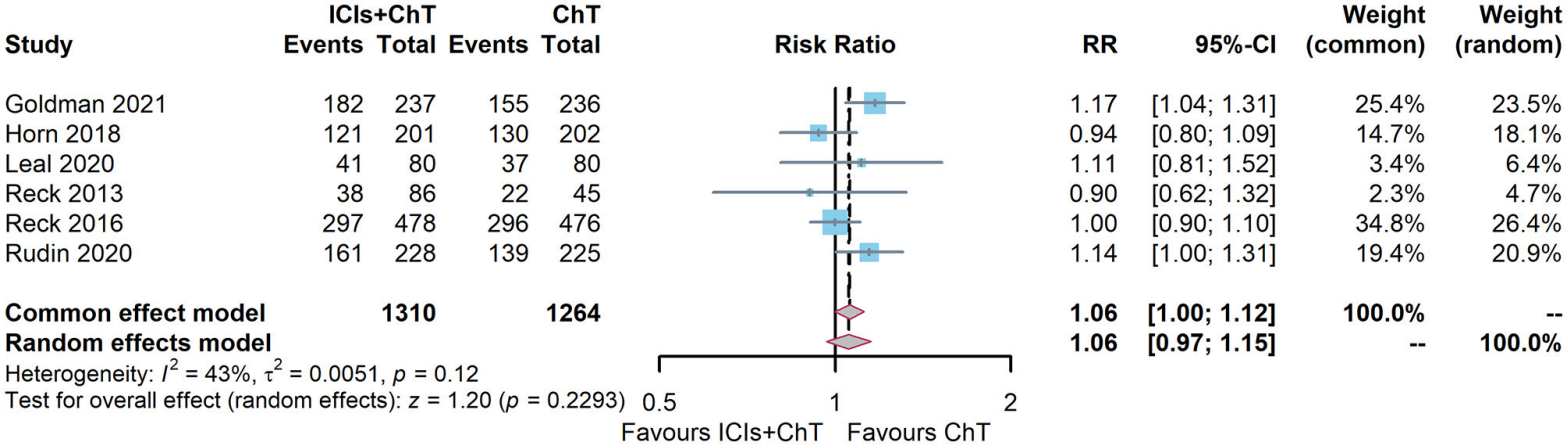
Comparison of objective response rate (ORR) between ICIs+ChT and ChT in SCLC.

Of the six included studies, five had reported the ORR of “ICIs + ChT” (6, 7, 10, 15, 16). The heterogeneity test showed that I^2^ = 43% and *P* = 0.12, indicating that the included studies were of good homogeneity, and the fixed-effect model should be used. Meta-analysis effect size of ORR was RR = 1.06, 95% CI = 1.00–1.12, and *P* = 0.06, indicating a trend of ascending ORR in “ICIs + ChT” compared with that in the chemotherapy alone, but the difference failed to be statistically significant (Figure 8).

**Figure 9:**
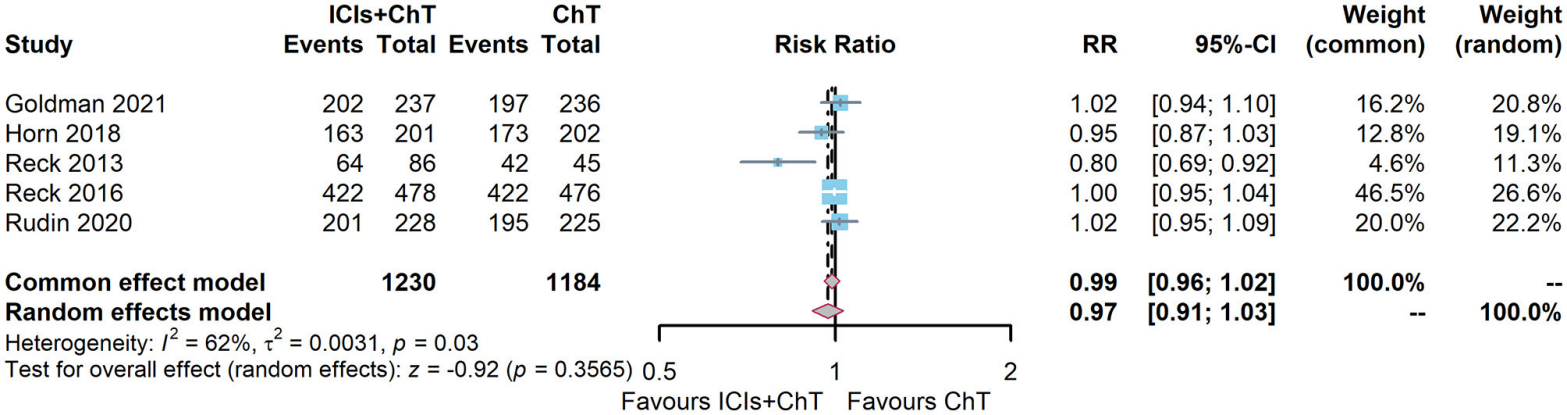
Comparison of disease control rate (DCR) between ICIs+ChT and ChT in SCLC.

DCR data on “ICIs + ChT” for SCLC were available in five of the six included studies (6, 7, 10, 15, 16). The heterogeneity test showed that I^2^ = 62% and *P* = 0.03, demonstrating that the results of each study were highly heterogeneous, and a random-effect model should be used. The meta-analysis effect size of DCR was RR = 0.97, 95% CI = 0.92–1.03, and *P* = 0.35, indicating that compared with chemotherapy alone, “ICIs + ChT” did not significantly increase the DCR (Figure 9).

### 3.8. Meta-analysis of adverse events

**Figure 10:**
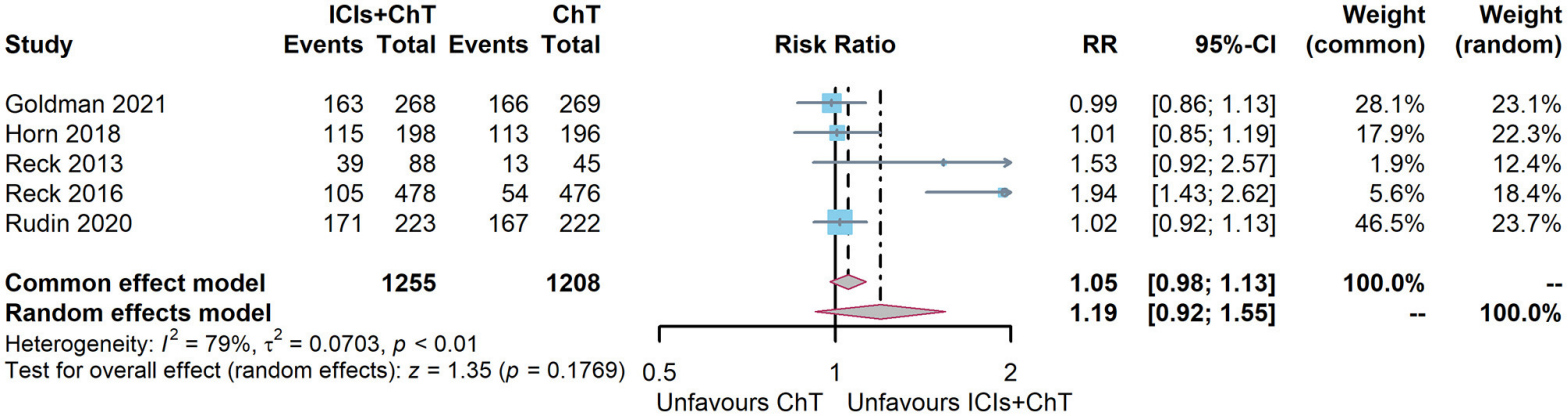
Comparison of grade 3–5 treatment-related adverse events between ICIs+ChT and ChT.

Of the six included studies, five had reported the incidence of trAEs (6, 7, 10, 15, and 16). The heterogeneity test indicated that *P* = 0.0007 and I^2^ = 79%, and the random-effect model should be used for combined analysis. The meta-analysis effect size of trAEs was RR = 1.16, 95% CI = 0.97–1.39, and *P* = 0.11, indicating that the incidence of trAEs of “ICIs + ChT” was similar to that in chemotherapy alone (Figure 10).

**Figure 11:**
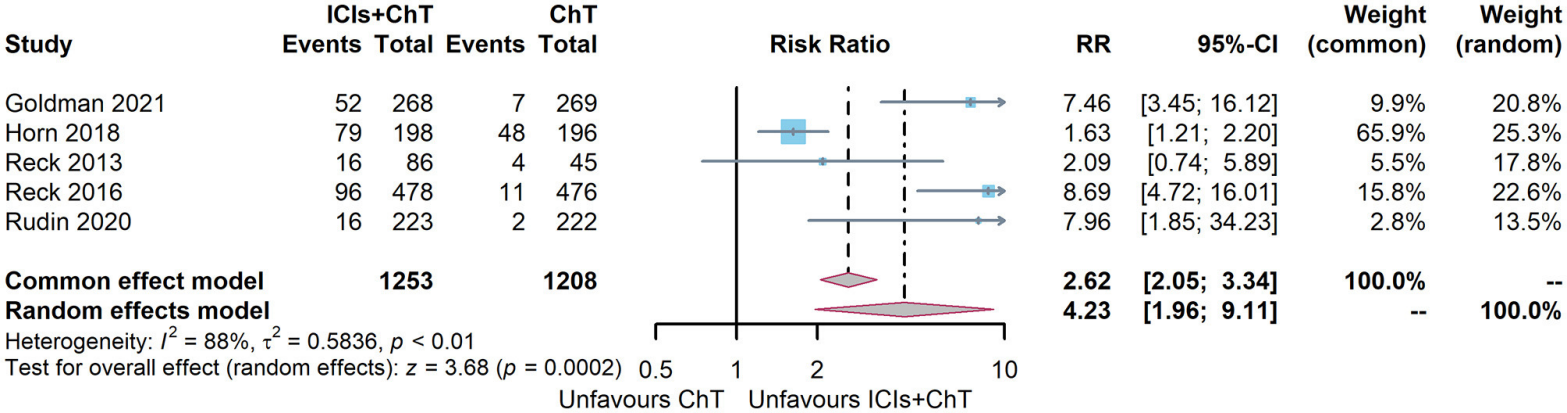
Comparison of grade 3–5 immune-related adverse events between ICIs+ChT and ChT.

Of the six included studies, five had compared the incidence of irAEs with “ICIs+ChT” and “ChT”. The heterogeneity test indicated that *P* < 0.00001 and I^2^ = 88%, and the random effects model should be used for combined analysis. The meta-analysis effect size of irAEs was RR = 4.29, 95% CI = 1.73–10.61, and *P* = 0.002, indicating that the incidence of irAEs in “ICIs+ChT” was significantly increased compared with that in chemotherapy alone (Figure 11).

**Figure 12:**
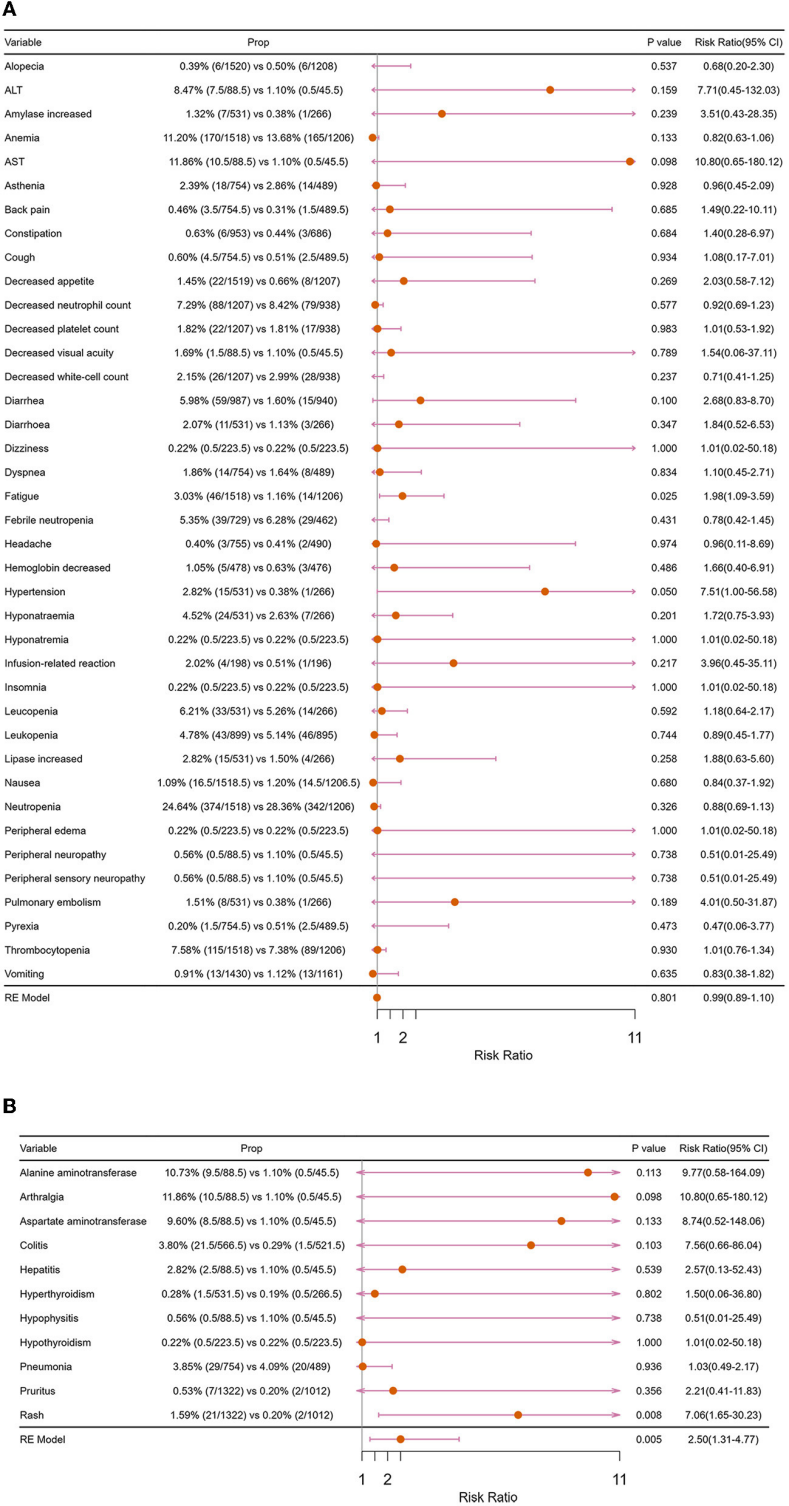
Forest plots of major adverse events: **(A)** treatment-related adverse events; **(B)** immune-related adverse events.

From the perspective of trAEs, the three most common trAEs of “ICIs + ChT” were neutropenia (26.64%), an increase in serum glutamic-oxalacetic transaminase (AST, 11.86%), and anemia (11.20%); however, compared with “ChT”, the incidence of these trAEs did not significantly increase (Figure 12A). From the perspective of irAEs, the three most common irAEs were arthralgia (11.86%), an increase in alanine aminotransferase (10.73%), and an increase in aspartate aminotransferase (9.60%). Although the incidence of single immune-related adverse events was not statistically significant, the combined incidence of irAEs showed significant differences (Figure 12B).

### 3.9. Analysis for bias and heterogenicity

**Figure 13:**
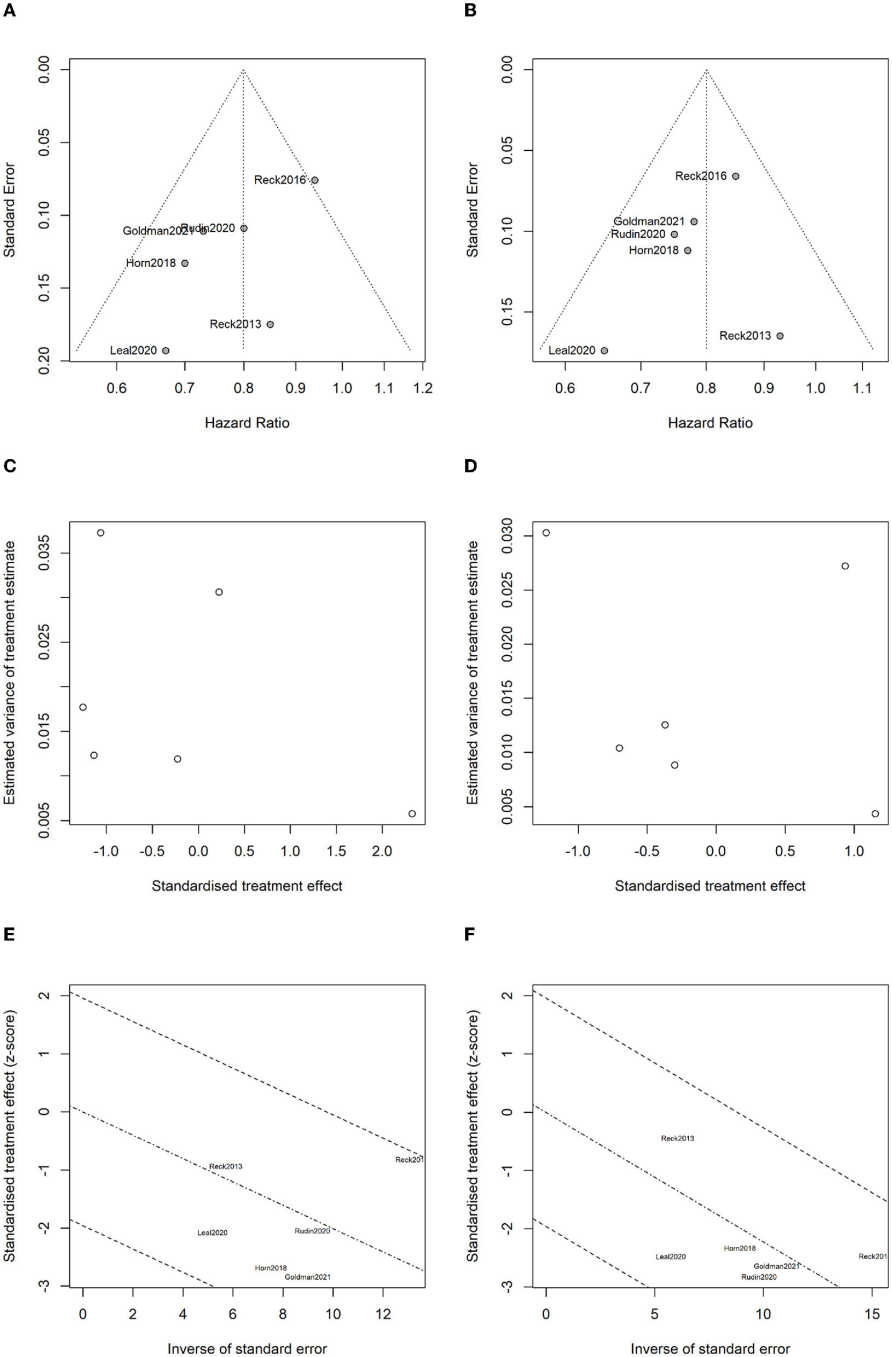
Analysis for bias and heterogenicity: **(A)** funnel plot of overall survival for evaluating publication bias; **(B)** funnel plot of progression-free survival for evaluating publication bias; **(C)** Begg's test results for overall survival; **(D)** Begg's test results for progression-free survival; **(E)** radial plot for overall survival to evaluate heterogenicity; and **(F)** radial plot for progression-free survival to evaluate heterogenicity.

The publication bias funnel plots for OS and PFS are shown in Figures 13A, B. Begg's test suggests that the funnel plots for OS (z = 0.75, *P* = 0.452) and PFS (z = 1.13, *P* = 0.260) were basically symmetric, indicating no publication bias (Figures 13C, D). Radial plots were used to evaluate heterogenicity, and the results are shown in Figures 13E, F. For both OS and PFS, all studies fell within the confidence interval of the radial plot, and the slope of the scatter plot is small, indicating no significant heterogenicity between different studies.

## 4. Discussion

The popularity of immunotherapy continues to rise, and it has become very effective in the field of tumor treatment, gaining widespread attention in various malignant tumors such as melanoma, kidney cancer, lung cancer, and colorectal cancer. An exploratory study called CheckMate 026 showed that tumor mutational burden (TMB) was considered a predictive biomarker associated with the efficacy of ICIs (18). A previous genome-wide analysis of 110 SCLC specimens found that the SCLC genome was unstable and demonstrated a higher TMB (19–21). Theoretically, SCLC has higher tumor neoantigens on the surface and is more susceptible to immunotherapy. Therefore, many scholars have explored the efficacy of ICIs on SCLC in recent years (22).

Ipilimumab, a CTLA-4 inhibitor and the first immune-targeted drug used in SCLC, preliminarily showed good clinical activity in the treatment of patients with extensive-stage SCLC in the CA184-041 study (15). The CA184-041 study is a randomized, double-blind, multicenter phase II clinical trial exploring the efficacy of ipilimumab in combination with paclitaxel and carboplatin (CP) as first-line therapy in ES-SCLC (15). The results of CA184-041 showed that compared with CP alone, CP followed by ipilimumab had significantly improved immune-related PFS (irPFS; HR = 0.64, *P* = 0.03), but did not prolong PFS (6.4 months vs. 5.3 months, HR = 0.93, *P* = 0.37) and OS (12.9 vs. 9.9 months, HR = 0.75; *P* = 0.13) (15). Subsequently, based on the CA184-041 study, a number of clinical trials on the treatment of SCLC with ipilimumab were carried out. The CA184-156 (NCT10450761) study was the only phase III study to date to explore the efficacy of ipilimumab as first-line therapy in the treatment of ES-SCLC (10). However, this study found that adding ipilimumab to etoposide plus platinum was not beneficial in improving OS in patients with ES-SCLC (10).

Despite disappointing data from the trials of ipilimumab in ES-SCLC, three large trials targeting the PD-1/PD-L1 pathway were subsequently conducted to explore the role of PD-1/PD-L1 inhibitors in ES-SCLC patients, namely IMpower133 (6), CASPIAN (7, 8), and KEYNOTE-604 studies (16). Unlike the CTLA-4 inhibitors, PD-1/PD-L1 inhibitors seem to have better performance in ES-SCLC. IMpower133 study was the first phase III RCT exploring the efficacy of atezolizumab combined with standard chemotherapy in ES-SCLC globally and demonstrated that atezolizumab combined with chemotherapy can significantly prolong OS and PFS in ES-SCLC (6). Based on the excellent results of the IMpower133 study, atezolizumab combined with Etoposide + Cisplatin (EP) had been recommended as the standard first-line treatment for ES-SCLC (6). CASPIAN study is a phase III RCT of durvalumab combined with standard chemotherapy in the treatment of ES-SCLC, and it had proved that this regimen can also significantly prolong the OS and PFS of ES-SCLC, and ORR as well (7, 8). KEYNOTE-028 study demonstrated the anti-tumor activity of monotherapy with pembrolizumab, a PD-1 monoclonal antibody, in previously treated patients with ES-SCLC (23). A pooled analysis based on KEYNOTE-028 and KEYNOTE-158 studies showed that the ORR of pembrolizumab alone was 19.3% in patients with relapsed or metastatic SCLC who had received ≥2 lines of prior therapy; the incidence of grade 3–5 adverse events was 9.6%, and 67.7% of patients had sustained remission for ≥12 months (24). KEYNOTE-604 study (NCT03066778) further demonstrated that pembrolizumab combined with standard chemotherapy can significantly improve OS and PFS in the first-line treatment of ES-SCLC (16). ECOG-ACRIN EA5161 is a phase II randomized controlled clinical study comparing the efficacy of nivolumab combined with standard chemotherapy in the first-line treatment of ES-SCLC (17). Although the full report of this study has not yet been published, in view of the positive results of nivolumab in this study, which has important clinical value, it is also included in this meta-analysis. This study also confirmed that nivolumab combined with standard chemotherapy in the first-line treatment of ES-SCLC can significantly improve OS and PFS.

Regarding the abovementioned studies, PD-1/PD-L1 inhibitors combined with standard chemotherapy can improve the efficacy of ES-SCLC. Although the abovementioned four RCTs found that ICIs combined with chemotherapy increased grade 3–5 irAEs, the overall safety and tolerability were acceptable, indicating that the regimen is safe and feasible. It can be seen from the six RCTs included in our study that CTLA-4 inhibitors and PD-1/PD-L1 inhibitors have different performances in the first-line combination therapy of ES-SCLC. However, there is no head-to-head comparative study comparing the efficacy of different PD-1/PD-L1 inhibitors as the first-line combination therapy in ES-SCLC. A recent network meta-analysis comparing the efficacy of atezolizumab, durvalumab, pembrolizumab, and nivolumab as the first-line treatment in patients with ES-SCLC found that atezolizumab, durvalumab, pembrolizumab, and nivolumab had no significant statistical difference in PFS or OS (25). However, durvalumab showed an ORR advantage compared to atezolizumab, but also a significantly higher risk of irAEs (25).

As demonstrated in the abovementioned RCTs, the combination of ICIs and chemotherapy as the first-line treatment for ES-SCLC is generally successful, and more and more ICIs have been approved, marking a new era of ICIs in anti-cancer treatment (26, 27). Our current study aimed to evaluate the efficacy and safety of ICIs combined with standard chemotherapy vs. chemotherapy alone in the first-line treatment of ES-SCLC *via* meta-analysis. At the same time, combined with the WHO-recommended Grades of Recommendations Assessment, Development and Evaluation (GRADE) system, an evidence-based evaluation of important outcomes was conducted, and possible treatment recommendations were elicited. A total of six RCTs were included in our study. Compared with chemotherapy alone, ICIs combined with standard chemotherapy as the first-line treatment in patients with ES-SCLC are more advantageous in prolonging OS and PFS. Apart from previously reported results (25, 28, 29), we presented the results of HR-based meta-analyses and mixed-effect model-based meta-analyses. The advantage of the mixed-effect model is that it makes full use of the survival information at different time points in the original study (14). Together with the sample size, it can achieve the same effect as individual patient data (IPD) meta-analysis, and the mixed-effect model can provide the estimated survival proportions at different time points; therefore, a combined survival curve can be drawn. As indicated in Table 2, the estimated 1- and 2-year OS rate in the “ICIs+ChT” and “ChT” group was 48.46% vs. 35.8%, and 15.84% vs. 10.12%, respectively. The estimated 6- and 12-month PFS rate in the “ICIs+ChT” and “ChT” group was 35.95% vs. 26.26%, and 12.23 % vs. 4.82%, respectively. A meta-analysis based on the mixed-effect model provided more direct survival information, and the survival curves for different treatments as well. Taken together, it can be concluded that compared with chemotherapy alone, ICIs combined with standard chemotherapy can reduce the risk of death in OS and PFS by 20% and 18.5%, respectively. However, no significant improvement in ORR and DCR had been observed. Only the CASPIAN study indicated that durvalumab combined with chemotherapy can significantly improve ORR (79.5% vs. 70.3%) (7, 8), while not in other studies. The underlying reason could probably be that SCLC was a chemotherapy-sensitive malignancy, and in the first-line treatment, chemotherapy could induce the greatest degree of tumor regression. Therefore, the combination treatment of ICIs could not significantly improve ORR and DCR. However, previous studies indicated that, unlike traditional treatments, immunotherapy can bring long-lasting immune responses and long-term survival benefits even after the patients had stopped using it, and in patients with stable disease (SD), which was known as the “smearing phenomenon”. The specific mechanism of the “smearing effect” remains unclear. One view is that immunotherapy has a unique mechanism of action in the anti-tumor process, which can initiate or restart the cancer-tumor cycle in patients, and amplify the immune effect, but not cause an unlimited autoimmune response. Based on this superiority of anti-tumor immune responses, immune memory may provide long-term immune protection, thereby enabling long-term survival (30). The smearing effect in the survival curves could be found in many clinical trials with immunotherapy. In CheckMate 017/057 studies, patients treated with nivolumab continued to show a long-term OS benefit at 5 years compared with those with docetaxel, with a 5-year OS rate of 13.4% vs. 2.6%. Regardless of the histology of squamous cell carcinoma or non-squamous cell carcinoma, patients treated with nivolumab were five times more likely to survive more than 5 years compared with chemotherapy. Therefore, immunotherapy had greatly prolonged the survival of patients with advanced-stage cancer (31, 32). From the perspective of PFS and OS benefits, although ICIs combined with chemotherapy cannot improve the ORR and DCR of patients with ES-SCLC, the smearing effect of ICIs can still enable these patients to achieve long-term survival. In addition to factors related to the tumor itself, DCR and ORR also have certain inherent limitations as evaluation indicators. ORR is the sum of the ratios of complete response (CR) to partial response (PR), as the direct measurement of tumor response to anti-tumor drug, which can reliably reflect the anti-tumor activity of the drug. However, a simple ORR is not enough to explain the problem, and sufficient duration of response (DOR) is also needed to evaluate the effectiveness of tumor treatment (33). The longer the duration of the response, the more likely it is that an increase in ORR will bring clinical benefits. DCR is the proportion of patients with stable disease (SD) on top of ORR. However, it is also susceptible to the natural course of tumors and cannot reliably reflect the anti-tumor activity of drugs (34, 35).

The safety of ICIs combined with chemotherapy is another major concern (36, 37). Understanding the possible AEs is of vital importance in the application of immunotherapy (37). In this study, we analyzed the differences in the incidence of grade 3–5 trAEs and grade 3–5 irAEs between “ICIs+ChT” and “ChT”. Compared with chemotherapy, “ICIs+ChT” as the first-line treatment in patients with ES-SCLC did not increase the incidence of grade 3–5 trAEs. However, the incidence of grade 3–5 irAEs was significantly increased. It has been reported that trAEs of ICIs were anemia, nausea, myalgia, decreased appetite, and neutropenia, while irAEs were rash, pneumonia, hepatitis, colitis, ophthalmia, and so on (38, 39). In the “ICIs+ChT” group, trAEs were more common in grades 1-2, and most of them could be alleviated with corresponding symptomatic treatment (38). According to previous reports, the incidence of grade 3–5 colitis and hepatitis in the “ICIs+ChT” group was higher than that in the chemotherapy group, and most irAEs could be controlled with a drug suspension and glucocorticoid administration (40, 41). In order to reduce the increased mortality caused by irAEs, the overall management principles are early detection, early evaluation, and early treatment (38, 42). It should be noted that approximately 10% of SCLC patients have paraneoplastic syndrome (PNS), and PNS in the nervous system is considered an autoimmune sequela. The presence of PNS may lead to a worse prognosis, and given the possibility of self-mimetic activation, attention should be paid to the increased incidence of PNS caused by immunotherapy (43). Fortunately, no significant increase in PNS was observed in patients receiving ICIs in combination with chemotherapy in IMPower133 or CASPIAN studies (1).

Compared with several previous meta-analyses on ICIs combined with chemotherapy vs. chemotherapy alone in the first-line treatment of ES-SCLC (25, 28, 29), the following advantages and innovations could be found in our articles: (1) the largest number of original studies were included, and two CTLA-4 inhibitor studies and four PD-1/PD-L1 inhibitor studies were also included. (2) Using innovations in meta-analysis methodologies, we not only adopted the traditional inverse variance meta-analysis method with HR as the effect value but also gave the results of the meta-analysis based on the mixed-effect model. We used the mixed-effect model proposed by Arends et al. to perform a meta-analysis on the survival proportions information in the survival curve (14) and granted the meta-analysis results in higher precision. We presented not only the forest plot but also the survival curves reconstructed by the meta-analysis, which was very valuable to the evidence users. (3) To the best of our knowledge, there is currently no evidence recommendation based on the GRADE system for ICIs combined with chemotherapy in the first-line treatment of ES-SCLC. In the GRADE system, although evidence based on RCTs is initially rated as high quality, confidence in the relevant evidence may be downgraded due to five important factors (44). According to the GRADE methodological quality evaluation, among the six indicators in this meta-analysis, four outcomes (OS, PFS, incidence of trAEs, and irAEs) were considered as high-grade evidence, while ORR and DCR were judged to be low-grade evidence. The main reasons for low-grade evidence are as follows: The full text of ECOG-ACRIN EA5161 has not been reported (17), and there is a lack of availability information of DCR, a risk of “incomplete outcome data” bias was assigned to the assessment; there is obvious heterogeneity between six studies, and the results of the meta-analysis are negative, limiting the generalization of the evidence. In addition, because the effect size is borderline, it is possible to change the statistical results of ORR by adding studies. Due to the need to sign the informed consent form for treatment, it is difficult to achieve double-blinding of patients and interventionists, so allocation concealment and blinding in this study were not used as an important evidence basis for consideration. In this study, we set OS, PFS, and the incidence of irAEs as the main “key” outcome indicators. Given the overall high quality of the original studies, the wide sample population with different ethnicities, and the obvious effect value of key indicators, the authenticity of the conclusions is reliable and the extrapolation is good. Therefore, we set the evidence recommendation level of these indicators as “strong recommendation” (1). However, as the summary effect value of trAEs was not significant, the level of evidence recommendation was set to be “weak recommendation” (2). Similarly, the level of evidence recommendation is set to be “weak recommendation” (2) due to the low level of evidence for ORR and DCR.

Immune checkpoint-blocking drugs represented by PD-1/PD-L1 antibodies have achieved surprising results in the treatment of various cancers, but the overall effectiveness is a key drawback (45). The heterogeneity of tumors and the diversity of the immune microenvironment are the main factors limiting efficacy (46). Inevitably, there is also a problem of drug resistance in anti-PD-1/PD-L1, which has attracted the attention of scholars (47). The combination scheme based on PD-1/PD-L1 antibodies is expected to address the shortcomings of low efficiency and susceptibility to drug resistance in a single target, making it a frontier in international research (47). With further research on the mechanism of signaling pathways of PD-1/PD-L1 and TGFR2/TGF- β in tumors, bifunctional anti-PD-L1/TGF-βRII agents have shown that it can simultaneously block PD-1/PD-L1 and TGFR2/TGF-β signal pathway, promote the activation of effector T cells, regulate the tumor microenvironment, reverse immunosuppression and fibrosis, and show better anti-tumor effect than PD-L1 monoclonal antibody in a variety of mouse tumor-bearing models (48). The bifunctional agent bintrafusp alfa (previously named M7824), comprising the extracellular domain of human TGFβRII (TGFβ Trap) linked to the C-terminus of the human anti-PD-L1 heavy chain (αPD-L1), has been developed in an attempt to address this issue (49). Recently, some novel anti-TGF-beta/PD-L1 bispecific antibodies such as YM101 (50) and SHR-1701 (46) have been developed, which effectively overcome anti-PD-1/PD-L1 resistance in some cold tumors (51). Anyway, immunotherapy has broad application prospects for small-cell lung cancer.

Although we had made a comprehensive summary of the existing studies, the following limitations still exist in our study. First, our analysis included only RCTs; however, phase III registration trials are often highly case-selective, and subjects in RCTs are not fully representative of real-world clinical patients. In RCTs, elderly and/or frail ES-SCLC patients, especially patients with comorbidities and worse performance status (PS ≥ 2), are often excluded from registration trials. Whether those patients can also benefit from ICIs treatment, no strong conclusions can be drawn (52). A current study based on real-world data suggested that the benefits of combination therapy with ICIs are comparable to clinical trials after adjusting for age and PS score (53). Whether ICIs are recommended for patients with pre-existing autoimmune disease, organ transplantation, or chronic viral infection (e.g., hepatitis B) is still debatable. For these patients, a multidisciplinary discussion should be adhered to decide whether to administer ICIs or not (54). Second, the studies included were all published in English. Third, the efficacy of ICIs may be related to some clinical factors such as gender, age, race, smoking history, and chemotherapy regimens. Our study did not further analyze the influence of these factors. Previously published meta-analyses had been conducted for subgroup analyses of these factors (25, 28, 29). Finally, as more and more ICIs are developed and applied clinically, it is still debatable whether these novel drugs can achieve the same clinical benefits as the existing ICIs.

## 5. Conclusion

Our meta-analysis has affirmed the clinical benefit of “ICIs+ChT” as the first-line treatment for patients with ES-SCLC with solid evidence. Compared with conventional chemotherapy, although “ICIs+ChT” had increased the incidence of grade 3–5 irAEs, ICIs combined with chemotherapy significantly improved OS and PFS in ES-SCLC patients, and their trAEs were acceptable. Therefore, ICIs combined with chemotherapy can be used as the first-line treatment for patients with ES-SCLC.

## Data availability statement

The original contributions presented in the study are included in the article/supplementary material, further inquiries can be directed to the corresponding authors.

## Author contributions

JZ and YD: conception and design and provision of study materials or patients. JZ and BH: collection and assembly of data. JZ and XC: data analysis and interpretation. JZ, YD, BH, and XC: manuscript writing and editing. All authors contributed to the manuscript and approved the submitted version.
